# Report on Smallpox in Calcutta, 1833-4, 1837-8, 1843-4, and Vaccination in Bengal, from 1827 to 1844

**Published:** 1845-10

**Authors:** 


### ART. V.

*Report on Smallpox in Calcutta,* 1833-4, 1837-8, 1843-4, *and Vaccination in Bengal, from* 1827 *to* 1844. By DUNCAN STEWART, M.D., Superintendent General of Vaccination. *Published by Order of Government.—Calcutta,* 1844. 8vo, pp. 284.

A certain measure of enthusiasm, a contempt of difficulties, perhaps an unwillingness to perceive them, are essential to the successful carrying out of any great and unaccustomed enterprise. Nor is this the case only in the busy scenes of life, but it holds good likewise in the more tranquil domain of science. Seldom, however, does enthusiasm indulge in brighter dreams than when science and philanthropy are hand in hand pursuing the same object. Of all the *idola* by which the mind of the philosopher is misled, none are so pardonable, none, we had almost said, are so respectable as those generated by the warm and loving heart of one who is labouring not for his own good or his own honour, but for the well-being and the happiness of all mankind. Never did scientific philanthropist enter on an investigation in which these fallacies were more likely to abound than in the pursuit of that discovery by which Jenner has immortalized his name, and blessed the world. A man of child-like simplicity of character, of most tender affections, of most ardent and untiring philanthropy, it might have been feared that his fancy would often lead his judgment astray; that the head would too readily give credence to all that the heart longed to believe. His early habits, however, had well fitted him for close and long-continued observation; his love of truth never wavered, and truth therefore never deserted him.

The lapse of time has nevertheless brought to light impediments to successful vaccination, and obstacles to its spread, of which Jenner could not but be ignorant. In our Indian empire especially, to which we owe so much, for which we have done so little, this great boon has been extended to comparatively few, though nearly half a century has elapsed since its introduction, and smallpox still rages there with undiminished frequency, and unmitigated severity.

"Reviewing," says Dr. Stewart, "the whole history of vaccination in Bengal, I fear it must be owned that its progress has been slow, that its operations have been but partially successful, and that its present state and prospects are unsatisfactory and discouraging. Neither can it be denied that public opinion has been unsettled regarding its advantages, and public confidence shaken in its efficacy and permanence as an antidote to smallpox, a feeling which is not confined to the common people, but prevails more perceptibly among the better informed and reflecting classes of the community, and is not without participators among the profession itself." (p. 261.)

Dr. Stewart's chief object has been to explain the causes of this inefficiency of vaccination in Bengal, and if possible to suggest a remedy. Besides this, he has given a description of three epidemics of smallpox which have prevailed at Calcutta since the year 1832, and has made known the results of his inquiries respecting a coincident epizootic malady which prevailed among horned cattle and some other domestic animals.

The epidemic of the year 1843 appears to have been the most severe of the three, and is that concerning which Dr. Stewart has collected the most extensive information. In each epidemic the ravages of the disease were nearly confined to the natives, and hence opportunities of observing its progress have been much fewer than they would have been in any city of England. The intercourse which medical men in India have with the natives is very slight, since the rich usually employ their own *hakeems*, and few of the poor, except those who are suffering from chronic affections, apply for relief at the dispensaries. Besides this, the superstition of the people nearly precludes them from bringing any case of smallpox to a doctor, since "it is an universal belief throughout the whole country that smallpox and measles are of supernatural origin, and that all medical interferences during their course are highly iniquitous, as they bring down the wrath of the Goddess from whose hands the scourge descends." (p. 221.)

Though the patients, however, do not often come under the care of European medical men, yet a tolerably correct estimate of the mortality from smallpox may be arrived at by an examination of the registry of native deaths kept at the different *Ghâts* and *Ghorastans*, where alone the funeral rites of cremation and interment are permitted to the Hindoo and Mussulman inhabitants respectively. From these it appears that the annual mortality in Calcutta from all causes has been about 5·11 per cent. during the past twelve years; that 4·2 per cent. of this mortality has resulted from smallpox: and that the total deaths from smallpox have been in the proportion of 1 to 458 living. Now, although this rate of mortality is more than four times greater than that which took place in England during the recent epidemic of 1839,* yet as the mortality from smallpox in London previous to the introduction of vaccination, amounted to 8 per cent. of the total mortality,† our first impression is naturally favorable, and we are inclined to suppose either that smallpox in India is less severe than in this country, or that a very decided influence has already been exerted on it by vaccination. Neither of these

* Second Report of the Registrar-general, Appendix, p. 17.

† Gregory's Lectures on the Eruptive Fevers. London, 1843. 8vo, p. 59.

inferences, however, would be correct. It appears that the bulk of the native population of Calcutta is migratory, consisting of labourers whose families live at a distance, and who remain in the city only until they have acquired a sufficiency to enable them to return to their own homes. "A peculiarity in consequence exists in the character of this population, which though startling is easily accounted for; it is *stationary* in amount, with a great excess of adults." This peculiarity must be borne in mind in any estimate that we may form of the fatality of smallpox in Calcutta. In England 75 per cent. of all the deaths from smallpox take place in children under 5 years of age. We find, however, from an examination of a table showing the ages at death, and the cause of the death of 20,000 native inhabitants of Calcutta, that only 33 per cent. of the deaths from smallpox took place in persons under that age. There seems to be no reason for supposing that infancy enjoys any peculiar immunity from this disease in India, and the discrepancy between this result and that arrived at by the Registrar-general can therefore be accounted for only by the small number of children in the population of Calcutta. If, however, the deaths from smallpox in Calcutta above the age of five years represent as in England only 2-9ths of the total mortality from that disease, we shall be forced at once to raise our estimate of the mortality it produces threefold, and to attribute 12·6 per cent. of all deaths in India to this fatal malady. Even allowing to the fullest extent for all possible sources of error in a calculation founded on such imperfect statistical information, it must nevertheless be manifest that the present mortality from smallpox in India, equals if it do not exceed that in London, at the close of the last century.

We could not describe the general features of smallpox in Bengal more clearly than has been done by Dr. Stewart in the following conclusions, the result partly of personal observation, partly of communications which he has received from medical men in different parts of the presidency:

"1st. All classes and casts of the natives, all ages and both sexes are pretty equally susceptible of infection, the mortality being mainly dependent upon the modifying circumstances of previous inoculation or vaccination, of natural constitution, of present health or feebleness of personal comfort or destitution affecting individuals; and by the particular constitution of the atmosphere, the salubrity of the locality, and the construction of the dwelling.

"2d. The great majority of the victims are totally unprotected either by vaccination or previous inoculation, though the latter practice is most common.

"3d. Those who have undergone the disease previously either naturally or communicated by inoculation, or who have been *successfully* vaccinated, always have the disease in a somewhat *modified* form. The incursive fever is often equally violent, but the eruptive stage is always milder, and the secondary fever proves fatal only in previously debilitated or scrofulous subjects."

Statements in the Third part of this Report, however, do not quite accord with this assertion as to the invariably modifying influence of vaccination.

"4th. In those who suffered from high fever at the onset with much cerebral and nervous excitement, headache, delirium, and severe lumbar pain, unless early destroyed by fever, the eruptive stage succeeds most fully and favorably, affording, though in a confluent form, great relief to the sufferings, and promise of a favorable termination. In these cases the chief danger arises about the 12th and 14th day from the secondary fever then occasioned by the erythematous condition of the skin.

"5th. The worst, and most certainly fatal cases, are those of asthenic type occurring generally in miserable, impoverished, and debilitated subjects, or in lethargic, scrofulous, and obese constitutions, in which early passive hemorrhages, the result of venous congestion, occur from the mucous surfaces of the bladder and bowels, or from the uterus in women, in which the maturative process is imperfect, and petechiæ or bloody vibices soon cover the skin, death ensuing about the 8th day from anemia, while the sensibilities are fully retained to the last moment.

"6th. The violence of the epidemic is moderated by a damp and hot atmosphere, its diffusion promoted by dry and cold weather." (pp. 50-1.)

The above-mentioned influence of season was remarkably shown in each of the three Calcutta epidemics. A few fatal cases occurred in each autumn; with the first cool day of November the disease became much more active, and the mortality continued to increase during the winter and spring. It remained unchecked by the heats of March and April, but was perceptibly diminished by the falls of rain in May, and disappeared totally on the establishment of the rainy season in July. The same rule governs the appearance and course of the measles and chicken-pox, and applies likewise to the activity of the vaccine virus which becomes almost extinct during the rainy season. Hooping-cough is the epidemic of that period, remittent fever abounds in the autumn, and cholera is always prevalent at the change of season, but especially at the setting in of the cold weather.

Another interesting fact is that the epidemic constitution which impressed its peculiar type and character on any epidemic of smallpox, likewise exercised considerable influence over whatever epidemic succeeded or replaced it. This was remarkably evident in the case of the epidemic cholera which succeeded the smallpox in May 1844, and which was distinguished by the same character of malignancy, and the same absence of attempts at reaction as had attended the former disease.

We now pass to the Second part of the Report, in which Dr. Stewart describes an epizootic disease that prevailed in Bengal in 1843-4. The opinion of the natives with reference to its nature is sufficiently shown by their employing to designate it the word *mattah,* which is one of the Bengalee terms for smallpox. This term, however, is applied, according to Dr. Stewart, very indiscriminately, and is not even restricted to diseases attended by an eruption of the skin. Whenever (as indeed often happens) any fatal epizootic malady appears among the domestic animals of a district, at the same time that some exanthematous epidemic is prevalent among the inhabitants, popular belief usually assigns to each of these diseases the same origin, nature, and name. On both of these accounts, therefore, Dr. Stewart was not surprised to hear in the autumn of 1843, that cattle were dying in great numbers from smallpox. The natives persisted in applying to it the name of mattah, notwithstanding the absence of any pustular eruption on the body of the animals. The symptoms generally observed were various indications of fever, such as panting, burning thirst, and loss of appetite, which were followed in a few days by violent purging, profuse salivation, and pituitary discharge from the nostrils. On the fourth day the tongue and palate became ulcerated, the animal began to pass bloody stools, and death usually occurred on the fourth or fith days. Sometimes death took place as early as on the third day; the mouth being parched, the eyes suffused, and the animal suffering from convulsive tremors.

"In a good many cases," says the author, "certain rough elevations were pointed out to me on the skin, having the hair turned back or ruffled over a small phlegmonous base. A number of these undoubtedly were produced by the burrowing of vermin or bites of insects, but many may also have been pustular without supposing them possessed of a specific character. As no possible good could be expected from experimenting upon human subjects with the matter from such pustules or ulcers, and the consequences could not be foreseen, I did not consider myself warranted in inoculating any children either from the flesh, matter, or the scabs which formed in any of these animals." (pp. 85-6.)

There were but three cases in which Dr. Stewart found papulæ, vesicles, or pustules on the teats or udders of cows; and in none of these cases were the characters of the pustule those of cowpox, nor was the inoculation of four children with the matter taken from them followed by any effect whatever. Both Dr. Stewart and Mr. Greenfield, an extensive cattle-dealer, who had observed the true cowpox in England, are of opinion that the disease did not present any resemblance to it. Dr. Stewart, however, goes on to state that it bore no resemblance whatever to any bovine disease with whose description he is acquainted. From observations so incomplete and details so scanty as those of Dr. Stewart, it is indeed not easy to determine positively the exact nature of the disorder; still, so far as the symptoms and course of the affection are described, they tally exactly with the accounts of the Pestis bovina, which committed such ravages among cattle in Europe during the course of the last century. Dr. Stewart's position in India, probably without access to books, explains his ignorance of these accounts. Had he been acquainted with them, they would have taught him that the pustular eruption on the skin of the animals was one of the distinguishing features of the European disease, while the appearance of vesicles or pustules on the teats or udder is very far from being of general occurrence. Inoculation of some healthy animals with the matter from these pustules would soon ascertain whether or no the disease be like the Pestis bovina communicable by contagion; but even this obvious experiment was not made. In the absence, then, of any satisfactory proof to the contrary, we are inclined to coincide in the correctness of the native opinion with reference to this distemper among cattle; regretting at the same time that Dr. Stewart should not have detailed the particulars of a single case of this disease, that he should not have made a single post-mortem examination; and that, notwithstanding the prevalence at the same time of a most fatal disease, regarded by the natives as smallpox, among domestic fowls, he should not have taken any pains to ascertain its real nature. But we gladly leave this, which is by far the least satisfactory part of Dr. Stewart's Report; especially as the third part contains much interesting information, and does credit alike to the industry and good sense of the author.

Many pages are taken up with an examination of the defects in the different schemes by which the Indian government has sought to promote vaccination, and by suggestions for carrying it out more effectually. Other causes, however, beside defective administrative measures, tend to prevent the diffusion of vaccination, and the consequent mitigation of smallpox. Most of these causes, though common to India generally, appear to exist in a greater degree in the presidency of Bengal than elsewhere. First, among the influences that retard the spread of vaccination, may be mentioned the prejudices of the natives, among whom "a partiality exists in favour of smallpox inoculation, founded on ancient usage, and a belief that this disease operates on the bodies of young children as a purifier from the uncleanness contracted during parturition." (p. 229.) That dread of change so strikingly characteristic of all eastern nations contributes not a little towards maintaining this prejudice. Mr. Tweedie, however, mentions in his communication a far more valid reason for the natives' preference of the old method to the new

"Variolous inoculation," says he, "as performed by the Brahmins, produces a much milder disease than in Europe; a moderate degree of fever, with very slight and often no eruption, I believe to be the usual result, and this is said to be effected by inserting the virus by a number of scarifications over a large surface, and using matter which is rendered less virulent by keeping." (Appendix, p. xx.)

We do not find this peculiar mildness of inoculated smallpox noticed by any other of the gentlemen whose communications appear in this Report. It is a point, however, deserving the most intelligent investigation, and the rather since there would be some reason for doubting whether vaccination in India ever affords the same amount of protection from smallpox which it confers upon the inhabitants of more temperate climates. Dr. Trench, of H. M. 49th foot, writes from Dinhapore that he

. . . . . . . "had succeeded in effectually putting a stop to the spread of smallpox in his regiment by extensive revaccination. A marked difference was observable in those who were born and vaccinated in India from those who had been vaccinated in their youth in England. In the latter the general character of the disease was that of modified smallpox; whereas all the fatal cases were found among those born and vaccinated in this country." (pp. 143-4.)

This again is the only statement of this kind contained in the Report; but evidence in abundance is given proving that almost always, in certain districts, and almost everywhere, at certain seasons, the vaccine virus becomes deteriorated, the vesicle assumes an altered appearance, and the disease can often not be perpetuated at all, even with these changed characters. Mr. Gerard writes from Sabathoo that

"During the course of the epidemic (of variola) he was obliged to discontinue vaccination, finding that the climate, at least in part of May and June, was against its success, several cases being instantly superseded by smallpox." (p. 141.)

The Superintending Surgeon at Cawnpore reported, in March 1830,

"Unfortunately at this season the experience of past years has shown that the lymph is *wholly inert* in this climate." (p. 142.)

Mr. Tweedie writes thus:

"Dr. Stewart remarks a point of great importance, viz., that throughout Hindostan the virus is either lost altogether or degenerates in its powers during the hot months, and again resumes its original appearance in the cold season; from which I am led to think that it is only when in full force, so as to affect the system with a febrile irritation, that it can be *depended on* as a prophylactic; and as native children are inferior in energy to European children, the disease is of a less satisfactory character, and the system is often unaffected—hence one principal cause of the want of confidence in vaccination among the natives." (Appendix, p. xx.)

The degeneration of the vaccine vesicle was so great in the year 1837 as to excite Dr. Stewart's apprehensions lest the prophylactic should be entirely lost, and a similar change, though to a less extent, has since been observed annually, from May to September.

"The vesicles at this time became extremely minute, the surrounding induration small, the areola diffuse and ill defined, the course of the disease hurried and unsatisfactory. Yet, strange to say, notwithstanding these unfavorable appearances, I have observed them uniformly to disappear on the approach of cool weather, and the disease resumes speedily in the month of November the perfectly-developed Jennerian character." (p. 153.)

But not merely do the physical characters of the vesicle change at certain seasons, but it is quite evident that at those seasons the potency of the virus greatly declines. In proof of this, we again quote Dr. Stewart, who writes thus, in the year 1841:

"During the three past years, particularly at holiday seasons, and during the hot weather and rains, I have experienced much difficulty in keeping up what I could consider a healthy and genuine pock. The charges of lymph taken in June, July, and August have almost invariably failed, and I have occasionally been obliged to defer forwarding any supply until September, or later, the cold weather appearing to be by far the most favorable time for propagating the virus in Bengal." (p. 157.)

This mutability in the characters of the vaccine vesicle presents a still more serious impediment to successful vaccination, from the fact that the changes do not always occur at the same season. Mr. J. M. Ston, superintendent of vaccination at Bombay, writes in a letter, dated March 14, 1841:

"I have in two successive years remarked the periodical alteration in the characteristics of the vaccine to which you allude. In Bombay it has invariably occurred in the hot months of April, May, and June, and not in the rains, at which season and in the cold months it was as perfect as we ever see it. . . . . . . . . "In the hot weather the proportion of failures to successfully vaccinated is very great, and I at one or two periods have been apprehensive that the disease might be lost. On reference to the records in office, however, I find that the experience of my predecessor accords with yours, and that in the rains of 1837, 'at the commencement of the monsoon, the vesicle was observed in all the subjects vaccinated to be considerably smaller than usual (about one half), and it continued to exhibit this decreased size without any other alteration in its character throughout the monsoon, but on the setting in of the cold weather the eruption resumed its ordinary appearance.' " (p. 158.)

Here then we have an instance of sudden change in the period at which the degeneration of the vesicle takes place; a change for which there seems to be no assignable reason.

The chief obstacles to the spread of vaccination in India, as we gather them from different parts of the Report (for Dr. Stewart seems but little skilled in arranging his facts, or in laying the conclusions to which they lead before his readers) may be classed under the following heads:

1st. Native prejudice.

2d. The propagation of a spurious disease owing to the carelessness of native vaccinators.

3d. The influence of climate, which for about six months in the year, renders the vaccine vesicle imperfect, and for three out of those six months so modifies the virus as usually to render vaccination altogether unsuccessful.

4th. The fact that this influence of climate varies much in different parts of India, coming into operation in some places as early as March, in others not till two or three months later; and the additional fact that a similar variation will take place at the same locality without any known cause.

5th. The existence of some constitutional peculiarity in the natives, which renders them indisposed to the reception of the vaccine virus, or at least interferes with the full development of the vesicle ;* and the circumstance that smallpox after vaccination more frequently occurs in a grave form in the case of the natives of India than of Europeans.

On the other hand the absence of any such prejudices against inoculation and the alleged fact of the great mildness of the disease so produced appear to us not only to warrant, but to demand an examination of the following points :

1st. The mortality from inoculated smallpox among the natives.

2d. The amount of protection afforded by inoculation and vaccination respectively, and the mortality in each case from secondary smallpox.

3d. The influence of locality, climate, and season upon inoculation as well as upon vaccination.

If it were ascertained that the mortality from inoculated smallpox in India is really very inconsiderable, and that the disease thus produced is far less severe than in European countries; if the protection afforded by inoculation be complete and lasting, while that resulting from vaccination is uncertain, imperfect, and transitory, if moreover inoculation be practicable during the whole of the year, while for six months vaccination is either impracticable or of doubtful efficacy, it would become a question what course would be most likely to confer lasting good on the population of India, whether efforts should not be directed towards the easier task of diffusing inoculation, rather than the more difficult one of extending the somewhat questionable boon of vaccination?

We trust that we shall not be misunderstood in these remarks, and that none of our readers will suppose that we introduced a panegyric on Jenner as prefatory to an attack on vaccination. Abundant evidence exists in this volume of the benefits which in many instances resulted from vaccination. Many cases are here recorded of the vaccinated escaping smallpox though the disease was raging around them, or of their suffering from it in a very modified form. It is true that we have not dwelt on the bright side of the picture, but we did so because it seemed to us that more good would be gained by contemplating its shadows. We do not anticipate that the result of a careful investigation would be to establish the truth of that which we have put hypothetically, we do not expect that inoculated smallpox would be found to be really a disease of so mild a character, or that the success of inoculation would be ascertained to be unaffected by celestial and telluric influences, such as modify so greatly the powers of vaccination. We think, however, that difficult though such an inquiry would be in a country like India, it yet ought to be made. They who have directed their efforts to promote vaccination in the East have assumed inoculation to be an almost unmixed evil. Facts have come to light which seem to render the truth of this assumption questionable. Further examination may show it to be perfectly correct: and if not? well, if not—it will yet be worth much to have exchanged uncertainty, for well-founded belief, even though the creed should be somewhat different from that which we have been wont to profess. *Amicus Plato, sed magis amica veritas.*

* The latter part of this statement has been illustrated by evidence in an earlier part of this article; as proof of the former part we subjoin an extract from a letter from Mr. Smith at Myseri. "Matter," says he, "taken from a small pustule in a native, and one that by no means appeared satisfactory, has almost invariably produced in the *European* child a perfectly formed large and healthy vesicle." (p. 159.)

